# The Promotion of Eating Behaviour Change through Digital Interventions

**DOI:** 10.3390/ijerph17207488

**Published:** 2020-10-15

**Authors:** Yang Chen, Federico J. A. Perez-Cueto, Agnès Giboreau, Ioannis Mavridis, Heather Hartwell

**Affiliations:** 1 Department of Food Science, University of Copenhagen, Rolighedsvej 26, 1958 Federiksberg C, Denmark; yangchen@food.ku.dk; 2 Centre for Food and Hospitality Research, Institute Paul Bocuse, BP25, 69131 Ecully, France; agnes.giboreau@institutpaulbocuse.com; 3 Department of Applied Informatics, University of Macedonia, 156 Egnatia str., 54006 Thessaloniki, Greece; mavridis@uom.edu.gr; 4 Faculty of Management, Bournemouth University, Fern Barrow, Poole BH12 5BB, UK; hhartwell@bournemouth.ac.uk

**Keywords:** digital interventions, behaviour change, eating behaviour, digital health, health promotion

## Abstract

Diet-related chronic disease is a global health epidemic giving rise to a high incidence of morbidity and mortality. With the rise of the digital revolution, there has been increased interest in using digital technology for eating behavioural change as a mean of diet-related chronic disease prevention. However, evidence on digital dietary behaviour change is relatively scarce. To address this problem, this review considers the digital interventions currently being used in dietary behaviour change studies. A literature search was conducted in databases like PubMed, Cochrane Library, CINAHL, Medline, and PsycInfo. Among 119 articles screened, 15 were selected for the study as they met all the inclusion criteria according to the Preferred Reporting Items for Systematic Reviews and Meta-Analyses (PRISMA) search strategy. Four primary digital intervention methods were noted: use of personal digital assistants, use of the internet as an educational tool, use of video games and use of mobile phone applications. The efficiency of all the interventions increased when coupled with tailored feedback and counselling. It was established that the scalable and sustainable properties of digital interventions have the potential to bring about adequate changes in the eating behaviour of individuals. Further research should concentrate on the appropriate personalisation of the interventions, according to the requirements of the individuals, and proper integration of behaviour change techniques to motivate long-term adherence.

## 1. Introduction

Eating behaviour is one health risk behaviour that can have a major effect on health and vitality. Poor nutrition may lead to chronic diseases such as diabetes, cardiovascular conditions, hypertension, osteoporosis, cancer, and even dental caries [1].

An ideal diet consists of whole grains, various fruits and vegetables, protein, oils, and fat-free or low-fat dairy products. Ideal eating behaviours include reducing the sodium content in food and avoiding solid fats, which are the primary sources of trans fatty acids and saturated fatty acids [2]. An unhealthy diet may result in caloric intake in excess of caloric expenditure, which may ultimately result in obesity. In children and adolescents, lack of proper nutrition may lead to decreased cognitive performance and other developmental problems [3]. Childhood obesity is detrimental to proper physical and mental health development in children, and may lead to health problems including early-onset puberty, respiratory conditions such as asthma, dermatological infections, predisposition to cancer, and development of type-2 diabetes [4]. Obesity also may affect mental health by contributing to psychological conditions including poor self-esteem, anxiety, eating disorders, and being overly conscious about body image [5,6]. Longer-term effects of childhood and adolescent obesity include an increased likelihood of adult obesity, lower life expectancy, and the compounding effects of additional comorbidities [7,8].

The UK is currently suffering from an obesity epidemic. According to reports from the National Health Services (NHS) England, 27% of adults living in the UK were classified as obese in 2016 compared to only 15% in 1993. From 2016–2017, ~617,000 patients admitted to the NHS had a primary or secondary diagnosis of obesity [9]. The UK has the highest incidence of adult obesity among all Western European countries, and the Organisation for Economic Co-operation and Development (OECD) report predicts that 50% of the UK population will be obese by 2050 if adequate prevention programs are not implemented soon [10]. Obesity is strongly associated with social deprivation, as the prevalence of obesity among children who drop out of school in economically disadvantaged areas was 24.7%, but was 13.1% in the least disadvantaged regions [11]. It is essential to implement effective strategies to slow down the drastic growth of the obese population.

### 1.1. Changing Eating Behaviour

The optimal strategy for addressing the rise in obesity and related chronic illnesses is to cultivate significant changes in the dietary behaviour of children and adolescents, as well as adults. Effective behaviour changes that are relevant to everyday life are challenging because these behaviours do not occur in isolation but are part of a complex system. Historically, knowledge of the nutritional value of food was believed to be sufficient to change dietary behaviours because researchers assumed that greater nutritional knowledge would automatically encourage people to consume a healthier diet. However, extensive studies of the available literature have shown that educating people about the health benefits of nutritious food is not sufficient to effect necessary dietary changes [12]. Behavioural science is useful for creating specific strategies that will be necessary for productive behavioural changes.

Mediating variables, which can be personal, physical, environmental, behavioural, or familial, are important in the formation of dietary behaviours [13]. The most effective way to install changes in dietary behaviour is to alter one or more of the mediating variables [13]. For example, most children readily eat fruits and vegetables that they are familiar with [14], which may limit the variety of choices available to them. By exposing children to a greater variety of fruits and vegetables by increasing availability at home that offer a greater variety of fruits and vegetables, that is, by changing the mediating variable “environment”, children are more likely to change their dietary behaviour and consume more fruits and vegetables [15,16].

Designing interventions to change dietary behaviour is a complex process, which includes identifying and prioritising all mediating variables, defining the types of change that are needed, and then implementing appropriate policies. Change cannot be achieved without sufficient qualitative research involving focus groups and exhaustive interviews with the target population [17]. Most strategies are currently based on intuition and the social, cultural, and economic characteristics of the target population rather than evidence-based interventions. Because different individuals may respond differently to the same message, additional research in the area of behavioural science is needed to develop strategic policies that will be effective for the majority of people in the community.

### 1.2. Digital Behaviour Change Interventions

In the modern digital age, many people are connected to the internet by various technologies. Between 20% and 80% of people use the internet to monitor their health and for other health benefits [18,19,20]. Therefore, digital interventions offer an ideal opportunity to engender necessary changes in dietary behaviour. A digital behaviour change intervention (DBCI) is defined as ‘…a product or service that uses computer technology to promote behaviour change’ [21]. These interventions are accessible through wearable devices, computer programmes, cellular phones, smartphone applications, and various websites. Designing DBCIs involves an interdisciplinary approach, which harnesses expertise from disciplines including computer science and behavioural science to develop a collective approach for engaging the target population. An effective DBCI is designed by connecting knowledge of computer programming, content of the intervention, design of the interface, and delivery by human–computer interaction [22].

Research shows that DBCIs can induce individual changes by inspiring people to lead healthier lives, potentially helping millions. DBCIs have been effective in facilitating health behaviour change for weight management [23], smoking cessation [24,25], increasing physical activity [26], decreasing alcohol consumption [27], and self-management of chronic conditions [28]. The effectiveness of DBCIs depends on a commitment to, and continual interaction with, the target population [29]. For example, websites and mobile apps have been developed to help people quit smoking, manage anxiety disorders, or ensure timely administration of medications. In many cases, these websites and mobile apps function as personal therapists by offering motivation, feedback, and knowledge in response to personalized lifestyle information from the patient. Thus, DBCIs may prove quite valuable for providing interactive support services to public health campaigns [30].

Despite the immense potential of DBCIs for implementing changes in dietary behaviour, important limitations include access to the internet using appropriate tools such as a smartphone or computer and the ability to utilise these technologies or spend time learning their operation.

Another major limitation is the time commitment required for digital interventions to be effective. Users must enter all data into the system, otherwise, the DBCI will be no more beneficial than static content such as printed material. Continuous updating of data by the user is a ‘hassle factor’ that can gradually erode the effectiveness of a DBCI [31] and result in people dropping out of online programmes as they lose motivation. Meanwhile, individuals need to use DBCI for a minimum amount of time to have any meaningful effect on behaviour, and users need to remain motivated during that time. As with any behavioural change, there is an opportune (teachable) moment when the user is most amenable to make behavioural changes or is at risk of a setback. Digital interventions are unable to identify and act appropriately on these moments, which decreases their efficacy. The introduction of sensor technologies can reduce the ‘hassle factor’, and devices such as GPS, accelerometers, and galvanic skin response (GSR) sensors may be able to identify the most favourable moments to effect behavioural change; for example, by gauging the moment when a user is susceptible to stress build-up and helping them take steps to de-stress [32].

### 1.3. Rationales behind the Study

Obesity and overweight are now considered leading risk factors for mortality [33]. Obesity and overweight may lead to cancers of the ovary, kidney, thyroid, breast, uterus, pancreas, gall bladder, stomach, oesophagus, bowel, liver, as well as myelomas and meningiomas. The prevalence of many cancers is increasing due to the rise in obesity [34]. According to the OECD, cancer causes ~222 deaths per 100,000 people in the UK, making the UK the 11th highest among all OECD countries in cancer mortality [10]. Of these cancer cases, 6% have been attributed to obesity [35]. Similarly, the number of caesarean births in the UK rose by 11% in five years to 28% in the year 2016–2017, and this unprecedented increase has been partially attributed to the rising prevalence of obesity, as 1 in 5 women have a BMI > 30. Nearly 25% of the total adult population in the UK are obese, which has been estimated to cost the NHS £ 14 bn per year. In the absence of effective action to address this situation, this amount is predicted to rise to £ 50 bn by 2050 [36].

Similar to adult obesity, childhood obesity in the UK has risen steadily in recent years. Reversing obesity in adulthood has proven to be exceedingly difficult; therefore, it is essential to develop programmes to prevent childhood obesity. Although various environmental, behavioural, and genetic factors are known to contribute to childhood obesity [37], behavioural and environmental factors likely play a predominant role [38]. Sedentary lifestyles, lack of physical activity, addiction to video games, television and smartphones, and rapidly transforming dietary behaviours are major factors contributing to the obesity epidemic [39]. The ease of availability and intensive marketing of high-calorie food products aid in increasing obesity levels, as seen in developing countries such as India, Mexico, and Brazil [40]. In developed countries like the UK, the high purchasing potential of consumers, the availability of inexpensive energy-rich food products, and the popularity of high-sugar-content carbonated drinks have accentuated the obesity problem [41]. In the absence of comprehensive structural changes, the already struggling NHS will be unable to cope with this epidemic.

In 2006, the UK Department of Health estimated that £187 bn were spent each year for treating preventable conditions [42]. In recent years, the realisation that money could be better spent on social campaigns for promoting healthy lifestyles has resulted in a government policy shift from curative to preventive medicine.

Public health promotion campaigns motivate the target population to change their behaviour using the reward and punishment (or incentive and disincentive) method, such as preventing alcohol abuse in young people by portraying alcohol consumption in a negative light. Several strategies, tools, and theories have been used to create these campaigns, which follow the 4Ps commercial marketing strategy to maximize effectiveness, and include: product, price, placement, and promotion [32]. Certain campaigns that focus on motivation and creating awareness of specific health issues provide special telephone hotlines or referrals to health care centres or other facilities to support early diagnosis and increase survival rates for potentially fatal conditions. Evidence suggests that health promotion social campaigns can influence ~5% of the population on average [43], but the percentage may differ depending on the population or behaviour being targeted. For many health-related behaviours, even small changes such as 5% in the behaviour of a population can produce considerable impacts on public health.

Although social campaigns may create awareness and motivate people to change their habits, certain behavioural changes such as substance abuse, addictions, smoking, mental health, and dietary changes are difficult to address. Convincing someone to initially adopt a healthy lifestyle has proven to be easier than maintaining motivation to continue practising healthy lifestyle behaviours. For certain behavioural changes, interventions must be tailored to personal lifestyle, tastes, habits, desires, time constraints, and location. However, social campaigns cannot create a personalised healthy diet according to personal tastes, needs, and preferences. In 2011, Cugelman et al. [44] demonstrated that although social campaigns have the ability to affect 5% of the population, digital interventions could affect behavioural change in 10% of the population. Advantages of the digital world in customisation and personalisation have more significant impacts in achieving difficult behavioural changes such as altering dietary habits. In addition to efficiency, digital interventions are more cost-effective than other types of personalised support. For example, a personalised digital anti-smoking intervention is the least expensive, but personalised print interventions cost 5-40 times more and personalised assistance by telephone is 150–250 times more expensive [45]. Thus, the efficacy, reach, and low cost of DBCIs make them ideal for effectuating dietary behavioural changes and improving public health.

In this paper, we reviewed the literature on digital dietary behaviour change interventions. The aims were to: (1) Compare different types of digital interventions available to the public; (2) Gauge the efficacy of each type of intervention on dietary behaviour changes by studying effect sizes; (3) Recommend policies and formulate strategies for further interventions.

## 2. Materials and Methods

This systematic review followed the guidelines of the Preferred Reporting Items for Systematic Reviews and Meta-Analyses (PRISMA) and has been registered with the International Prospective Register of Systematic Reviews (PROSPERO; registration number CRD42019120085).

### 2.1. Search Strategy

Information sources were obtained from a literature search of the electronic databases PubMed, CINAHL, PsycInfo, MedLine, and Cochrane Library using the search terms ‘digital interventions’, ‘smartphone apps’, ‘diet’, ‘digital’, ‘behaviour’, and ‘dietary behaviour changes’ with a search filter restricted to English. Query results were downloaded and then combined and sorted to remove duplicates using the reference management tool EndNote X7.0.1 (Clarivate Analytics, Philadelphia, PA, USA). Since digital interventions only started appearing in recent years, there was no restriction on publication time.

### 2.2. Inclusion and Exclusion Criteria

The criteria for article selection were: (1) Full-length articles published in peer-reviewed journals; (2) Articles describing all types of clinical trials, randomized controlled trials, and other trials; (3) Use of a digital intervention to change dietary behavior; (4) Sample population consisted of healthy adults, children, or adolescents.

The criteria for article exclusion were: (1) Reviews, incomplete trials, and studies where only abstracts were available; (2) Sample population suffering from illness or disorders such as diabetes, cardiovascular disease, depression, or other mental health conditions; (3) Studies where a diet or digital intervention was present but were not present together.

### 2.3. Data Extraction and Analysis

**Figure 1:**
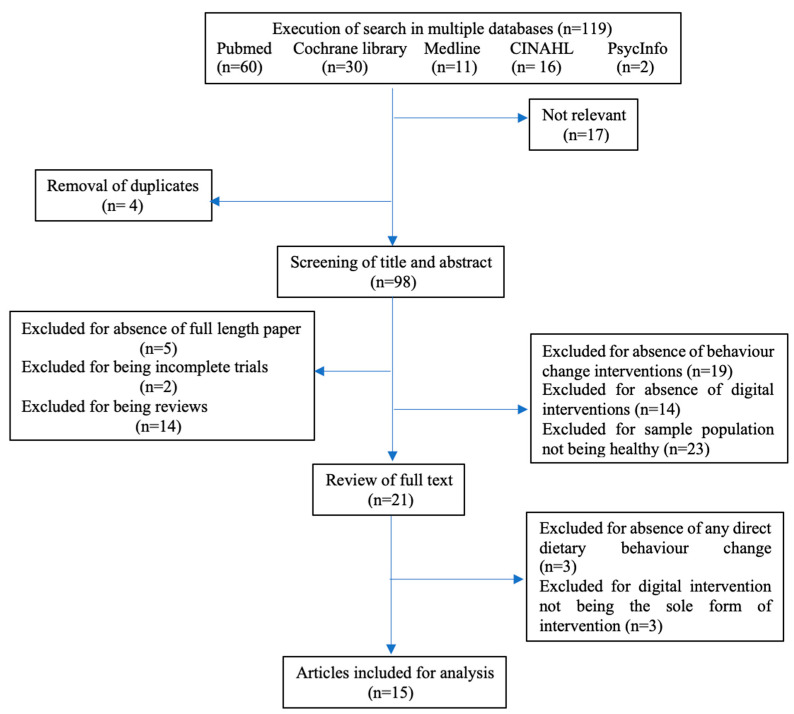
Flow diagram showing the Preferred Reporting Items for Systematic Reviews and Meta-Analyses (PRISMA) strategy used to search the literature.

Data were extracted and analysed using the PRISMA guidelines. The flow diagram of the search strategy is provided in Figure 1. Table 1 lists the articles that were selected for the review. Effect sizes used in this review were extracted from each study when available, or were calculated from available information (means and standard deviations, *t*-test *p*-values, and correlation coefficients) if not specifically reported using the formulae described by Lipsey and Wilson [46]. Two reviewers independently performed data extraction from September to December 2019. Quality assessment was further analysed by a third reviewer and finalized by consensus. Due to the limited number of articles and high heterogeneity among studies, a narrative synthesis was performed.

### 2.4. Quality Assessment

Risk of bias was assessed by two reviewers independently, then the agreed assessment was further entered into the Risk of Bias (RoB) tool [47]. If agreement was not achieved, a third reviewer would contribute to the assessment. Other discrepancies were resolved by discussion among the reviewers. There are six domains for quality assessment: (1) Randomization process; (2) Deviation from intended interventions; (3) Missing outcome data; (4) Measurement of the outcome; (5) Selection of the reported results; (6) Overall bias. Each judgement has three options: low risk, some concerns, and high risk.

**Table 1:** Characteristics of the articles selected for analysis.

Author, Year	Target Population	Intervention Type	Eating Behaviour Change	Effect Size
**Personal Digital Assistant (PDA) as intervention**				
Acharya et al., 2011 [48]	192 people with a mean age of 49 years and BMI of 34.0 kg/m^2^	Self-monitoring PDA	Increased consumption of fruits, vegetables and decreased intake of refined grains	Effect size for fat intake was 0.25; for fruit servings 0.36; vegetable servings 0.32; whole grain servings 0.1 and refined grain servings 0.2
Ambeba et al., 2015 [49]	210 overweight adults (BMI ≥ 34.0 kg/m^2^)	Daily tailored feedback on diet intake using a PDA	Significant improvements in intake of fats and carbohydrates	Effect size calculated between groups receiving feedback versus not receiving feedback (i) 0.32 for reduction of fat intake (ii) 0.34 for reduction of energy intake
Burke et al., 2010 [50]	Healthy adults (18–59 years of age) with a BMI between 27 and 43 kg/m^2^	Self-monitoring diet and exercise using a PDA with or without feedback	Higher proportion of the group using PDA and feedback had a significant weight loss (5%) after 6 months by monitoring calorie intake in their diets	An effect size of 0.3 in change in total fat intake was observed between the paper record group and group using PDA + feedback
Atienza et al., 2008 [51]	27 healthy adults aged ≥ 50 years	PDA monitoring their daily diet, providing feedback and answering questions	Target population reported higher intake of vegetables and dietary fibre in their daily diet	Effect size of 0.9 for vegetable serving and 0.7 for dietary fibre intake was calculated
Olson et al., 2008 [52]	Adolescents visiting 5 rural primary care practices in the USA	PDA-mediated questionnaires, health behaviour assessments and counselling	Increased intake of milk	Effect size of change in milk intake between the PDA group and non-PDA group was 0.365
**Online education as intervention**				
Schwarzer et al., 2017 ^#1^ [53]	454 adults (18–65 years of age)	Online platform delivering a lifestyle intervention following Mediterranean diet	Overall improvements in Mediterranean diet	Various effect sizes on dietary behaviour were observed; R^2^ = 0.14 for positive outcome expectancies; R^2^ = 0.12 for dietary action control; R^2^ = 0.13 for dietary planning and R^2^ = 0.17 for stages of changes
Kattelmann et al., 2014 [54]	1639 college students	10-week intensive intervention focussing on eating behaviour, physical activity, stress management via e-mail and the internet	Small changes were observed in fat intake and inclusion of fruits and vegetables in the diet	Effect size of fruit and vegetable consumption between control and experimental group was 0.05
O’Donnell et al., 2014 [55]	Students from 8 participating institutions in the USA (18–24 years of age), BMI ≥ 18.5	10 online lessons with feedback, facts and interactive questions	Setting of goals increased intake of fruits and vegetables by the participants	Effect size of fruit intake before goal setting and after 10 weeks of goal setting is η^2^ = 0.09
Grimes et al., 2018 [56]	Child–parent dyads from 5 government schools in Australia	5-week intervention programme delivered weekly via an online education programme to reduce salt intake	Increased knowledge, self-efficacy and behaviours related to salt in children but no reduction in salt intake was observed	An effect size of 1.08 was reported in change in dietary behaviour pre- and post-intervention
**Video games as intervention**				
Zurita-Ortega et al., 2018 [57]	47 university students, average age 22.53 years	12-week intervention by active video and motor games	Quality of diet was improved	Effect size of diet change post intervention versus pre-intervention was 0.68.
Shiyko et al., 2016 [58]	47 healthy, highly educated women, average age 29.8 years, average BMI 26.98	Computer game called Spaplay with real world play patterns and linked to real-life activities like healthy snacking	60% of participants were contemplating, 34% were preparing to and 4% demonstrated nutritional behaviour change	Effect size of nutritional knowledge gain was 0.86
**Smart phone applications (apps) as intervention**				
Duncan et al., 2014 [59]	301 adult male participants age 35–54 years	IT based 9 month intervention called ManUp influencing dietary behaviour and physical activity	Increased intake of high fibre bread and low-fat milk	Effect size was 0.07 for low fat milk intake and 0.2 for high fibre bread intake
Ipjian and Johnston, 2017 ^#2^ [60]	30 healthy adults, average age 34.4 + 15.7 years, average BMI 25.3 + 4.3 kg/m^2^	App called MyFitnessPal aiding in reduced sodium intake	Those using the app reported lower urinary sodium levels	Effect size for the study was reported as η^2^ = 0.234
Mummah et al., 2017 ^#3^ [61]	135 overweight adults 18–50 years of age, BMI 28–40 kg/m^2^	Vegethon mobile app enabling setting goals, self-monitoring and feedback	Significant increase in daily vegetable consumption in the intervention group	Effect size Cohen’s d = 0.18 for primary outcome measures after the 8-week trial and d = 0.2 for 24 h recalls
Wharton et al., 2014 [62]	57 healthy adults 18–65 years of age, BMI 25–40 kg/m^2^	Use of ‘LoseIt!’ diet tracking app	Weight loss was similar across groups using the app, memos or papers; healthy eating habit values decreased for app users; more app users completed the trial	Effect size of healthy eating index was 0.089

^#1^ = Effect size represented as R^2^ which is based on the variance; ^#2^ = Effect size represented as η^2^ which is the ratio of the variance; ^#3^ = Effect size represented as Cohen’s d, which is the difference between the experimental and control mean divided by a standard deviation for the data.

## 3. Results

Following screening, 15 articles were selected for inclusion in this review. During the screening process, care was taken to include studies that demonstrated or targeted a direct change, or intent to change, and eating behaviours of the sample population. Most studies investigated the effect of the interventions on other behaviours, such as physical activity and self-monitoring. Studies in which changes in dietary behaviour were also responsible for weight loss in the experimental groups were preferred over studies in which weight loss was attributed to regular exercise or other behaviour changes. Although various intervention strategies employed digital media, the selected articles covered four broad categories: use of a personal digital assistant (PDA), use of smartphone applications, online education or web-based intervention, and use of video games.

### 3.1. PDA

In 2008, Atienza et al. [51] observed in a randomised trial that use of a PDA compared to written nutritional, educational material precipitated a significant change in dietary behaviour. The intervention group was exposed for eight weeks to a hand-held device that monitored their intake of vegetables and whole grains twice daily and provided individualised feedback and support to help establish goals. The intervention group demonstrated an increase in consumption of vegetables and dietary fibre with a high effect size. Using the ‘Healthy Teens counselling approach’, Olson et al., [52] found that a PDA could assist clinicians in implementing dietary interventions. In this study, a screening tool based on a PDA was used as an office intervention at primary care centres involved in adolescent health behaviour counselling. The 6-month intervention resulted in an increase in milk consumption and physical activity. PDA use by adolescents reduced time spent by staff and clinicians in making individual reports and enabled clinicians to address sensitive issues concerning eating disorders.

Feedback is essential in a PDA-based intervention. Several studies have demonstrated improvement in interventions that have used a PDA coupled with feedback. In 2011, Acharya et al. [48] reported an increase in fruit and vegetable intake and a decrease in fat and refined grain consumption in a group exposed to a PDA compared to a group using paper records (PR). The software programme used by the PDA provided immediate feedback to the user regarding nutrient content and calories ingested, so that individuals could modify their next meal to meet daily nutrition targets. This approach resulted in greater adherence to the programme and attainment of goals. In the PR group, self-monitoring was conducted manually, and the time commitment increased with the number of target nutrients. The nutrient database included in the PDA made it easier for users to learn about the nutrition content of food; whereas, participants in the PR group expended greater effort to acquire knowledge about appropriate food choices. In addition, the portability and social acceptability of a PDA made it easier to use in any setting or environment.

In another study, Burke et al. [50] observed greater weight loss by a group using a PDA coupled with feedback compared to groups using only PR or a PDA. Secondary analysis of the Self-Monitoring and Recording using Technology (SMART) trial confirmed the importance of daily feedback in a PDA-based intervention. Ambeba et al. [49] reported a reduction in calorie and fat intake over a 24-month period when participants received remotely-sent daily feedback. Effect sizes for an increase in fruit and vegetable intake and a decrease in fat and energy were low to moderate (0.1–0.3) in most studies, except that of Atienza et al., a small study (*n* = 27 adults) where effect sizes were higher (0.7–0.9). A comparison of these articles is presented in Table 2.

### 3.2. Online Education

Web-based intervention or nutritional education has been observed to positively affect different age groups. A study involving 1639 college students revealed that a 10-week intensive web-based intervention, consisting of 21 short lessons and regular e-mail messages called ‘nudges’, increased fruit and vegetable intake in the intervention group [54]. Although no net change in BMI or weight was observed, this positive dietary behaviour change had an effect size of 0.05. A study by O’Donnell et al. [55] explored the advantages of setting goals and the effects of web-based interventions on changing dietary behaviour of students. The intervention consisted of 10 online lessons with data for weekly goals and behaviour. Intake of fruits and vegetables increased significantly with time in the intervention group (*p* < 0.001) with a moderate effect size (η^2^ = 0.09). Elapsed time also significantly affected goal attainment (*p* < 0.001). Schwarzer et al. [53] investigated the effects of a lifestyle intervention delivered by an online platform, which addressed four psychological constructs to change dietary behaviour in adults. Using a Mediterranean diet adherence screener, participants who had lower expectancies of positive outcomes gained more from the intervention. Grimes et al. [56] studied the influence of a web-based salt reduction programme (DELISH) in children on knowledge, attitude, and behaviour regarding daily salt-intake. The trial tested child–parent dyads and observed increases in knowledge of high salt foods and changes in salt-related dietary behaviour, with high effect sizes of 1.16 and 1.08, respectively. However, actual salt intake was unchanged. A summary of the results of these studies is represented in Table 3. The overall effect sizes in these web-based interventions were relatively low, but the high effect size 1.08 [56] for increase in nutritional knowledge supported the importance of the internet for disseminating knowledge.

### 3.3. Video Games

The effects of a 12-week video game-based intervention consisting of active and motor video games targeted at changing dietary behaviour were examined in university students [57]. Students displayed moderate adherence to a Mediterranean diet, but individual nutrients were not measured. Another trial targeting adult, highly educated women showed a high effect size (0.86) for nutritional knowledge [58]. The intervention consisted of a social online video game known as ‘SpaPlay’, which was developed to cultivate healthy eating and exercise in women. Participants had the option of personalising and individualising the content, which resulted in a longer-lasting impact. Effect sizes in these studies for nutritional knowledge and dietary change were high (0.6–0.8; Table 4).

### 3.4. Smartphone Apps

A randomised controlled trial (RCT) known as ‘ManUp’, which examined the effects of a web-based and mobile phone-based intervention in changing dietary behaviour in middle-aged men, was delivered to participants by print (control group) and information technology media (intervention group) [59]. Change in consumption of dietary fibre and milk from baseline to 3 months showed a low effect size; however, this change was not sustained throughout the study and no differences were observed between the intervention and control groups.

Another RCT involving overweight adults exposed to the ‘Vegethon’ mobile app over a 12-month period demonstrated an increase in vegetable intake by the intervention group with an effect size of 0.18 [61]. This was the first study to examine the effects of a standalone mobile app, which incorporated theory-based information and feedback/goal setting, on dietary behaviour change. High rates of adherence in this study suggest that participants were motivated to lose weight because they had already enrolled in weight loss programmes.

A small pilot study of a mobile app called ‘MyFitnessPal’ examined sodium intake of 37 healthy people [60]. A moderate to high effect size for decrease in urinary sodium levels was observed in the intervention group, suggesting that smartphone apps are capable of potentiating dietary behavioural changes. In an 8-week weight loss trial, Wharton et al. [62] reported that smartphone apps were better suited for self-monitoring than improving dietary quality. In this study, three participant groups using (1) a memo only, (2) paper and pencil, or (3) the mobile app ‘LoseIt!’ were followed to assess self-monitoring of daily food intake. The memo group and the paper/pencil group also received counselling, while the app group received feedback but no counselling. Results indicated significant weight loss, excellent self-monitoring, and a higher rate of trial completion by the app users. However, healthy dietary behaviours decreased over time in the app group, but did not decrease in the other two groups, indicating the importance of counselling in instilling eating behaviour change. Although effect sizes for this intervention ranged from low to medium, sodium intake was significantly reduced in two studies [60,62]. The results of these studies are summarised in Table 5.

### 3.5. Study Quality Assessment

**Figure 2:**
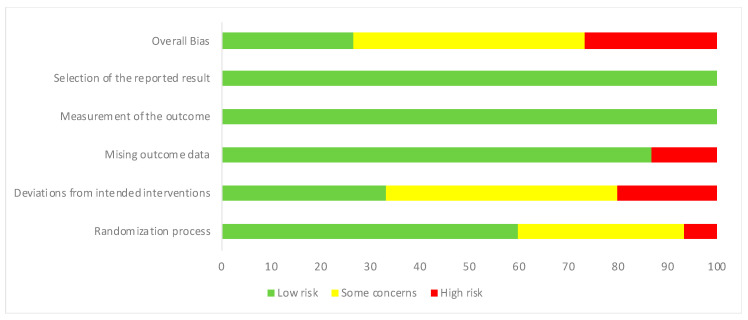
Risk of bias graph: risk assessment across all included studies.

Figure 2 shows the risk of bias across all included studies. All studies were classified as low risk in the “selection of the reported result” and “measurement of the outcome” domains. Two studies [54,55] were considered high risk in the “missing outcome data” domain, as outcome were only measured for part of the participants randomized in the studies. Three studies [52,55,58] were rated high risk in the “deviations from intended interventions” domain due to lack of appropriate analysis (e.g., sensitivity analysis) to estimate the effect of assignment to intervention which has a potential substantial impact on the results. Those rated as some concern in the same domain [51,53,54,56,57,60,62] also lack appropriate analysis, but without a potential substantial impact on the results. All studies rated as some concerns in the “randomization process” [56,57,58] were not fully randomized while the one rated high risk [52] were also not fully randomized, and the baseline differences between intervention grouped suggested a problem with the randomization process.

## 4. Discussion

A sedentary lifestyle, combined with a diet rich in calories and added sugar, have resulted in the emergence of obesity as a major health problem that is globally associated with increased mortality and morbidity, as well as an increased prevalence of chronic diseases [63,64,65]. The number of obese people in the world is predicted to increase to 3.3 billion by the year 2025 [65]. A high prevalence of overweight or obesity negatively impacts the economic health of many countries, accounting for ~7% of all health care expenditures throughout the world [66]. In 2010 alone, a high BMI was associated with 3.8% of disability and 3.4 million deaths worldwide [67]. Therefore, maintaining a healthy weight is extremely important and research has shown that even small amounts of weight loss can result in a considerable reduction in mortality, morbidity, and health care costs [68,69,70,71]. Loss of 1 kg of body weight has been shown to reduce the risk of developing diabetes by 13% [72]. Lifestyle interventions required for weight management include improved dietary quality, increased physical activity, and a restriction in the number of calories being consumed.

In the modern digital age, digital interventions are becoming increasingly popular for effecting lifestyle changes. This review presented various studies in which digital interventions have been used to produce dietary behaviour changes. The 15 articles selected for analysis concentrated on specific categories of interventions and studies that described direct effects on dietary behaviour were included. An overall effect size was not calculated because parameters of change in dietary behaviour were not identical in all studies; however, individual effect sizes for each trial were calculated and reported. Use of a PDA coupled with personalised feedback appears to have had the optimal effects on implementing dietary changes. In general, all interventions showed improved results when coupled with counselling and feedback. Adherence to the programme was a significant issue in all trials. Hence, it is necessary to employ interventions that sustain interest and motivation and support participants through each step.

A major component of the dietary behaviour change technique (BCT) is routine self-monitoring of daily diet [73,74] because it increases awareness of the food being consumed and helps elucidate impediments to positive dietary behaviour changes. Once these obstacles are identified, corrective actions can be taken to correct them which will help in achieving the goal of a better dietary quality [75]. The conventional and most common manner of self-monitoring is recording daily dietary intake and other observations using a diary or memo, which is then presented to the interventionist weekly or once every two weeks for feedback. However, over time participants find it difficult to sustain this routine of self-monitoring [76]. Thus, directive and supportive feedback may be reduced when self-monitoring is discontinued. Maintaining a PR also entails manual calculations and finding information regarding the nutrient value of foods, which requires time and becomes tiresome for the user [74]. Substitutes for the paper record, such as a PDA and an online diary have become popular. Studies indicate that PDAs have been used to record food intake [77] and other data such as total amount of energy consumed in 24 h [78]. In 2004, Glanz et al. [79] revealed that PDAs consisting of dietary software programmes can be successfully employed for self-monitoring and consequently help in attaining established goals for dietary behaviour change.

Advances in technology have given rise to devices originally known as Personal Information Management (PIM) gadgets, which gave access to a portable collection of data [80]. These devices have evolved into PDAs that now include ‘smart’ watches, ‘smart’ pagers, internet appliances, cellular phones, palmtops, pocket computers, tablet PCs, hand-held computers, and wearable computers [81]. State-of-the-art devices are equipped with networking, voice recognition, and wireless capabilities, which increase their value in the healthcare industry. Although PDAs are rapidly replacing the antiquated PR system for storing data, PDAs have limitations. Despite the immense potential to improve dietary quality, simplify self-monitoring and increase programme adherence, using a PDA may prove difficult for the elderly and those with low literacy. However, these difficulties may be overcome with help from the interventionists.

Internet delivery of web-based behaviour change interventions focusing on smoking cessation and weight loss has been reported in a number of studies [82,83,84]. Virtual interventions have recently become popular for disseminating health information [85]. Studies included in this review found that internet use significantly increased nutritional knowledge of the target population [56], but interventions must be customized to the target population. For example, as college students are known to spend on average 6.5 h per day on the internet [86], web-based interventions are more likely to be successful in this population [54,55].

Interactive video games combined with external counselling provide an effective intervention capable of increasing programme adherence while maintaining motivation and support. Video games should be targeted to the participants based on characteristics such as interest in specific types of games or maturity and technical skills required for use [57,58].

The ubiquitous presence of mobile apps makes them a cost-effective form of intervention that is readily accessible by all participants at any time, and can be personalised, deliver feedback, and maximise interaction to increase effectiveness [87]. However, internet-based interventions should include counselling in order to sustain beneficial effects [62]. The recent increase in the number of health-promotion apps appears to have been beneficial to users [88], though evaluation of these apps in RCTs is still in progress and it will be necessary to incorporate theory-based behaviour change strategies [89,90,91]. Besides, it is important to take other mediating variables (e.g., social and environmental variables) into account while designing DCBIs, as they also contribute to individual behaviour change [13]. However, even with the social and environmental considerations, it is still hard to achieve sustainable behaviour change via DCBIs, if the environmental context is not optimised. For example, it is unlikely to adopt a healthy diet if the availability and accessibility of healthy food are low. Consumer-led changes and advancements in the food industry and government policies are required to provide a healthy and sustainable food environment [92].

There were several limitations of the study. Almost half of the studies included were rated as “some concerns” in the “overall bias” domain in the quality assessment, mainly due to biases in the randomisation process and lack of appropriate analysis to estimate the effect of assignment to intervention. In future, this limitation could be eliminated by restricting inclusion criteria with more precise outcome statistics, although that could require an extension of publication period to increase the number of studies available, considering the limited number of studies found in this review. Another limitation is that grey literature was not included in this review due to the level of skills required to make accurate interpretation. This may have contributed to a decrease in outcomes of the review and also to a skewing of the outcomes by not providing unpublished information, leaving the review vulnerable to publication bias. Finally, the results of the review could not be generalised to all digital dietary behaviour interventions because of the several populations excluded from the study (e.g., populations suffering from illness or disorders such as diabetes, cardiovascular disease, depression, or other mental health conditions). Due to the differences in the required dietary management (e.g., type of diet, intervention method), the evaluation of digital dietary interventions on these populations should be addressed separately.

## 5. Conclusions

An integral part of dietary interventions in weight management is the participant’s role in self-monitoring, goal-setting, and awareness of their dietary nutritional value. This review found that any type of intervention should be combined with appropriate feedback and counselling in order to sustain the desired effects. Many trials reported that delivery of personalised feedback messages in real-time was critical for supporting self-regulation during the weight loss programmes [49,50,51,52]. A study by Spring et al. [93] reported that when a diet-tracking system employing a PDA was used in conjunction with a telephone-mediated counselling programme, weight loss was significantly greater over a 12-month period than a counselling programme alone. Thus, future research on other lifestyle changes such as sleep quality and hygiene should focus on use of mobile technology to deliver tailored feedback messages.

Furthermore, as structural factors run hand in hand with behaviour factors in the global obesity crisis, more research effort on relevant structural factors is needed to strengthen the existing behaviour change interventions. It is also essential to explore the comparative efficacy of digital dietary behaviour change interventions in settings with or without adequate structural changes (e.g., public policy on unhealthy food marketing).

Digital interventions have the advantages of being (i) convenient to use, (ii) available in many geographic locations to a large number of people, (iii) customizable, (iv) sustainable (keeping users motivated and adherent to the programme), and (v) cost-effective. Of all these characteristics, customization, sustainability, and cost-effectiveness require further development. The Transtheoretical Model of Behaviour Change [94] proposes that an intervention will be most successful when it matches the behaviours and cognitions of the participants. Personalisation of health behaviour interventions according to the readiness of each individual will help place that person on a trajectory that leads to behavioural change. The human genome sequence and the recognition that genetic composition may influence health and diet has opened new opportunities for personalising interventions based on genetics [95]. Finally, adherence to, and sustainability of, interventions require additional study. One strategy that may increase adherence and sustainability is to promote social interactions between participants [96].

Thus, future research should concentrate on developing scalable and sustainable digital interventions that are tailored to the target population and integrate effective BCTs.

## Figures and Tables

**Table 2:** Summary of results from studies using a Personal Digital Assistant (PDA) as a digital intervention for dietary behavior change.

Article	Summary of Results	Limitations	Strengths
Atienza et al., 2008 [51]	Greater intake of vegetables per 1000 kcal and increased fibre consumption from grains in the PDA group	Small sample size, self-reported dietary intake, absence of generalisation to middle aged and older populations and low retention rate	First RCT to study the effect of a PDA in dietary behaviour change
Olson et al., 2008 [52]	Use of a PDA among teens resulted in increased milk intake; clinicians found PDA helpful in providing necessary counselling	Lack of precision in recall measures may have obscured dietary changes; height and weight were not measured	Use of a PDA helped clinicians in counselling, confirming the role of tailored counselling and monitoring in weight management
Acharya et al., 2011 [48]	PDA group exhibited higher consumption of fruits and vegetables and lower intake of refined grains compared to the PR group; self-monitoring combined with PR reduced intake of total fat, saturated and mono-unsaturated fatty acids	Lack of extrapolation of findings to a wider population than the homogenous, predominantly white, educated, full-time employed female population studied	Comparison of PR and PDA system of interventions along with self-monitoring and a 91% rate of participant retention after 6 months
Burke, et al., 2010 [50]	Self-monitoring and median adherence were higher in the PDA group than the PR group; PDA group had reduced fat and energy intake after 6 months; PDA+FB group demonstrated highest percentage of weight loss	Only 15.2% male representation in the population; only 6 months of follow-up data were presented	First large RCT studying PR, PDA and PDA + FB with a 91% retention rate
Ambeba et al., 2015 [49]	Daily feedback (DFB) group exhibited significant decrease in total fat and energy intake compared to no-DFB group after 2 years, supporting the necessity of feedback	Fewer males, inclusion of participants of particular ages and BMI range and reliance on self-reported dietary intake	Daily, tailored and automated feedback in real time in an ethnically diverse population studied for 2 years with a high retention rate

**Table 3:** Summary of results in studies using online education as a digital intervention for dietary behavior change.

Article	Summary of Results	Limitations	Strengths
Kattelmann et al., 2014 [54]	Experimental group reported small increase in fruit and vegetable intake but increase was not maintained at follow up; no decrease in weight but greater planning was observed in the intervention group	Self-selected attrition rates, self-reported eating measures and physical activity	Intervention content was individually tailored to increase adherence, satisfaction and confidence in the intervention
O’Donnell et al., 2014 [55]	Goal-setting using online intervention increased intake of fruits and vegetables; goal-setting was effective for behaviour change but not for maintenance	Goal-setting functions were not assessed; options for goal-setting were limited; self-reporting and choice of a healthy population	One of the few studies where goal achievement was linked to dietary behaviour change
Schwarzer et al., 2017 [53]	Significant change to Mediterranean diet; individual psychological preferences and readiness should be considered for an intervention	Lack of control group and randomization; self-reported dietary intake; self-selected participants; no attempt to compare cultural eating habits of different countries	First study to examine effects of online education on 4 social-cognitive constructs and study person-specific effects of interventions
Grimes et al., 2018 [56]	No change in salt intake but increase in knowledge about high salt food, salt efficacy and behaviour were improved in children	Lack of a control group, small sample size from one region and self-reporting	Study confirmed that web-based educational programmes can increase awareness and knowledge

**Table 4:** Summary of results in studies using video games as a digital intervention for dietary behavior change.

Article	Summary of Results	Limitations	Strengths
Shiyko et al., 2016 [58]	Nutritional knowledge increased significantly; participants in the action stage of behaviour showed superior effects; need for individualised games; shorter activities were preferred to ones with a longer commitment	Small exclusive group already motivated to lose weight, self-report, and lack of follow-up and a control group	One of a few studies to investigate the effects of video games on BMI and nutritional knowledge
Zurita-Ortega et al., 2018 [57]	Decrease in fat mass and a shift toward a Mediterranean diet was observed post-intervention; the problematic effect of video games was not improved	Lack of control group; study limited to university students	Demonstrated the potential of video games in weight management

**Table 5:** Summary of results in studies using smartphone apps as a digital intervention for dietary behavior change.

Article	Summary of Results	Limitations	Strengths
Duncan et al., 2014 [59]	Increased consumption of low-fat milk and high fibre bread in both print and IT groups after 3 months; intake returned to baseline levels after 9 months	Very low retention rates, limited number of observations, weight loss was not measured, and use of print materials could not be assessed	Study of IT and print based interventions in men
Wharton et al., 2014 [62]	Paper, memo and app group participants lost weight but dietary self-monitoring was highest in the app group	App users may have used other methods for weight loss; feedback given only in the form of calories consumed	Among the few studies that have revealed that smartphone apps can act as good self-monitors
Ipjian and Johnston, 2016 [60]	Greater adherence and significant decrease in urinary sodium levels in the app group; body weight remained unchanged	Use of two different data analysis techniques, no direct comparison of sodium intake over time, diet instructions differed between app and print groups and weight loss not observed	Smartphone apps monitoring individual nutrients can effect dietary changes
Mummah et al., 2017 [61]	Participants demonstrated high engagement with the app; a significant increase in vegetable consumption and weight loss after 8 weeks; outcome linked to frequency of app usage and individual participant characteristics	Lack of longer follow-up and generalisation of findings to a larger population	Theory driven nature of the app, goal-setting and self-monitoring resulted in greater adherence; substantial sample size, randomised controlled study design, validated FFQs * and 24-h recalls

* = Food frequency questionnaires.
